# Explainable CatBoost–TabPFN Hybrid for Mode-Resolved Interlaminar Fracture Toughness Prediction in Fiber-Reinforced Polymer Composites

**DOI:** 10.3390/polym18212621

**Published:** 2026-10-23

**Authors:** Md Arifuzzaman, Sayeed Rushd, Muhammad Ali Martuza, Mohammod Hafizur Rahman, Md. Kamrul Islam, Ayed Eid Alluqmani

**Affiliations:** 1 Department of Civil and Environmental Engineering, College of Engineering, King Faisal University, Al-Ahsa 31982, Saudi Arabia; 2 Department of Chemical Engineering, College of Engineering, King Faisal University, Al-Ahsa 31982, Saudi Arabia; 3 Department of Computer Engineering, College of Computer, Qassim University, Buraydah 51452, Saudi Arabia; 4 Chemical Engineering Department, College of Engineering, Imam Mohammad Ibn Saud Islamic University (IMSIU), Riyadh 11432, Saudi Arabia; 5 Department of Civil Engineering, Faculty of Engineering, Islamic University of Madinah, Al-Madinah Al-Munawara, Prince Naif Ibn Abdulaziz, Al Jamiah, Medina 42351, Saudi Arabia

**Keywords:** fiber-reinforced polymer composites, interlaminar fracture toughness, delamination, tabular foundation model, TabPFN, CatBoost, explainable artificial intelligence, SHAP, Bayesian optimization

## Abstract

Fiber-reinforced polymer (FRP) composites offer high specific strength, high stiffness, and good corrosion resistance, which makes them suitable for aerospace, automotive, marine, and wind-energy structures. Their interlaminar fracture behavior is harder to pin down because delamination testing is slow and costly and the response shifts with loading mode. This study presents an explainable hybrid machine learning framework for interlaminar fracture toughness under Mode-I, Mode-II, and Mixed-Mode loading. A weighted ensemble pairs Categorical Boosting (CatBoost), a gradient-boosting method, with the Tabular Prior-Data Fitted Network (TabPFN), a pretrained transformer built for tabular data. A Tree-structured Parzen Estimator (TPE) tunes the CatBoost hyperparameters and the ensemble weight against specimen-grouped cross-validation. The work draws on the public D-STANDART experimental dataset and treats DCB, ELS, and MMB as three separate prediction problems for G_IC_, G_IIC_, and G_C_ rather than pooling them into one model. The analyzed D-STANDART specimens comprise pultruded carbon-fiber-reinforced polymer (CFRP) material based on Zoltek PX35 carbon fiber. The data are partitioned at the specimen level, so no specimen contributes records to more than one subset, and feature selection uses the training data alone. SHapley Additive exPlanations (SHAP) then measure how each input shifts the predicted toughness in each mode. The framework reached an R^2^ of 0.9280 for DCB, 0.9870 for ELS, and 0.9627 for MMB, with RMSE values of 5.1759, 1.5041, and 2.8809 and MAE values of 1.6502, 0.5372, and 0.7000, respectively. Bringing a tabular foundation model into delamination analysis, and keeping the three loading modes apart, lets the framework report mode-specific feature behavior instead of one averaged picture.

## 1. Introduction

The high specific strength, stiffness, corrosion resistance, and low weight of fiber-reinforced polymer (FRP) composites make them suitable for aerospace, automotive, marine, civil infrastructure, and energy applications [1]. However, the mechanical behavior of these composites depends strongly on the fiber orientation, material characteristics, loading conditions, manufacturing parameters, and interlaminar damage [2]. Delamination and fracture under Mode-I, Mode-II, and Mixed-Mode loading are particularly detrimental to structural reliability [3]. Testing every material and loading condition experimentally is costly, slow, and difficult [4]. Reliable data-driven prediction of mechanical and fracture responses is therefore becoming increasingly important. Machine learning (ML) offers an efficient way to learn complex nonlinear relationships between composite parameters and experimentally observed responses [5]. An explainable ML framework can also identify the parameters that most influence the mechanical and failure responses [6]. An accurate and interpretable prediction framework can therefore support the efficient design, evaluation, and failure analysis of FRP composites [7]. Wan et al. [8] investigated the mechanical properties of sustainable engineered geopolymer composites using sodium carbonate activators. Zou et al. [9] examined the effects of Cu addition on the microstructure, mechanical properties, and fracture behavior of Al-Mg_2_Si composites.

Several ML techniques [10] have been used to predict the mechanical behavior of composite materials, including Linear Regression (LR), Support Vector Regression (SVR), Random Forest (RF), Extreme Gradient Boosting (XGBoost), Light Gradient-Boosting Machine (LightGBM), and CatBoost [11]. These methods have been used to predict tensile strength, elastic properties, fracture toughness, delamination properties, and other mechanical responses [12]. More recent tabular models, such as TabPFN, add another option for learning complex relationships in structured datasets [13]. Hui et al. [14] investigated the experimental and mathematical characteristics of multi-mode vortex-induced vibration in long-span bridges. Optimization methods such as Bayesian optimization (BO) have also been used to select ML hyperparameters [15]. However, some ML models cannot capture the highly nonlinear interactions between composite and experimental parameters. Conventional ensemble models are also sensitive to hyperparameter tuning, and a single model may not perform well across different fracture conditions [16]. In addition, most existing studies focus on prediction accuracy and offer limited interpretation of the influence of specific materials or experimental variables on predicted responses [17]. Bi et al. [18] developed machine learning approaches for predicting Mode-II fracture energy in interleaved CFRP composites. Other studies report global performance but do not assess Mode-I, Mode-II, Mixed-Mode, material-specific, or loading-specific responses separately [19]. Yan et al. [20] explored simultaneous improvements in the tensile properties and fracture toughness of bulk metallic glass composites. He et al. [21] evaluated thermal camera sensitivity and spatial resolution for detecting internal defects in CFRP using laser thermography. The novelty of this study is an explainable, mode-specific CatBoost–TabPFN hybrid framework that predicts FRP interlaminar fracture toughness under Mode-I, Mode-II, and Mixed-Mode loading. The framework integrates training-only feature selection, specimen-level data partitioning, TPE-based Bayesian optimization, and SHAP-based interpretation to provide a leakage-aware and physically interpretable prediction strategy. Leave-one-specimen-out validation and controlled baseline comparisons further strengthen the assessment of predictive robustness and generalization.

To address these limitations, this study proposes a hybrid machine learning framework for FRP composites based on CatBoost and TabPFN (PyTorch). CatBoost and TabPFN are combined in a weighted ensemble, and the parameters are tuned with the Tree-structured Parzen Estimator (TPE) algorithm. Feature selection uses the training data only to prevent leakage, and the mode-wise predictions of G_IC_, G_IIC_, and G_C_ are interpreted with SHAP.

### 1.1. Research Objectives

1. Develop an explainable hybrid machine learning framework to accurately predict the mechanical performance and fracture responses of FRP composites under Mode-I, Mode-II, and Mixed-Mode loading.
2. Use the publicly available D-STANDART experimental FRP dataset, with its double cantilever beam (DCB), end-loaded split (ELS), and mixed-mode bending (MMB) fracture test data, to predict G_IC_, G_IIC_, and G_C_.
3. Integrate CatBoost and TabPFN through weighted hybridization, and optimize their model parameters and contribution weights using TPE-based BO.
4. Apply SHAP to identify the most influential FRP material and experimental parameters and to determine their positive or negative contributions to the predicted fracture responses.

### 1.2. Research Scope and Novelty

This study developed an explainable machine learning framework to predict the mechanical and fracture properties of experimentally tested FRP composites under DCB/Mode-I, ELS/Mode-II, and MMB/Mixed-Mode loading. The proposed framework combines a weighted CatBoost–TabPFN hybrid, training-only feature selection based on Pearson correlation and mutual information, BO-based selection of parameters and ensemble weights, and SHAP-based interpretation. Its main novelty is the joint use of advanced tabular ML, leakage-free optimization, mode-wise fracture response prediction, and explainability for G_IC_, G_IIC_, and G_C_. Most existing studies, in contrast, rely on single models or examine isolated fracture properties.

### 1.3. Research Organization

The paper is organized as follows. Section 1 introduces the background, motivation, objectives, scope, and novelty. Section 2 reviews related studies and states the research gap. Section 3 presents the proposed methodology. Section 4 describes the dataset and experimental setup. Section 5 reports the results, including model comparisons, optimization, and SHAP explanations, and Section 6 discusses them. Section 7 concludes the study.

## 2. Literature Review

Previous studies show the strengths of data-driven techniques but also their shortcomings for composite fracture and delamination problems. Cakiroglu [12] and Navas-Pinto et al. [16] demonstrated the applicability of machine-learning models for predicting composite mechanical and fracture-related responses. In another study, an artificial neural network (ANN) model was developed to predict the thermal response from machining process parameters and thermographic measurements. However, that work focused on the thermal response during machining rather than on mechanical properties or delamination resistance, and it did not propose a hybrid model for predicting FRP mechanical behavior and failure responses. Brayek et al. [22] predicted the tensile performance of carbon fiber/epoxy composite laminates using machine learning approaches. Their ML models predicted the stress–strain curves effectively. However, the investigation was limited to tensile performance and did not address fracture toughness or delamination. Berladir et al. [23] predicted and optimized the mechanical and tribological properties of thermoplastic polymer composites with various fillers. However, their research did not consider FRP fracture toughness, delamination, or failure response. Roy et al. [24] investigated a melt-and-trap process for producing high-strength flexible composites from incompatible waste streams. He et al. [25] investigated the transition from sawtooth to brittle fracture behavior in cubane architectures under dimensional constraints.

Karabulut [26] used machine learning to predict the performance and failure properties of concrete beams reinforced with glass fiber-reinforced polymer (GFRP) bars. Several ML regression models were trained on experimental three-point bending results to predict beam deflection and load-carrying capacity. Zhang et al. [27] analyzed the three-dimensional dynamic response of infinite plates on multilayered orthotropic foundations under moving loads. Song et al. [28] investigated hydrogel-stabilized riverbank sand using NMR and CT technologies at multiple scales. Dong et al. [29] used machine learning to predict the residual strength of lightning-damaged carbon fiber-reinforced polymer (CFRP) laminates. They combined a U-Net damage recognition model with a neural network prediction model, using damage images, layup details, and lightning conditions as inputs. Tanase et al. [30] reviewed the use of machine learning to predict and optimize the performance of bio-based FRPs. The review covered applications of ANN, support vector machine (SVM), and tree-based techniques in material property prediction and composite development. However, it did not develop a framework for predicting the fracture toughness or delamination of FRPs. Prada Parra et al. [31] studied ML-based estimation of the mechanical properties of continuous FRP composites. However, their research addressed tensile behavior, not fracture toughness, delamination, or Mixed-Mode behavior. Zheng and Huang [32] examined interfacial reinforcement and tribological performance in resin-matrix composites reinforced with modified coconut-shell fibers. Yang et al. [33] investigated energy characteristics during progressive shear failure of rock joints and evaluated associated brittleness behavior.

Alhusban et al. [34] reviewed the use of ML methods for estimating the performance of FRP-reinforced concrete structures. They considered several supervised ML techniques for estimating flexural, shear, confinement, compressive, and bond strength. However, the review did not consider the fracture toughness or delamination characteristics of FRP composites. Wang et al. [35] investigated active wellbore cooling using microencapsulated phase-change materials during deep drilling circulation. Mohammed et al. [36] used ML to predict the tribological properties of polymer nanocomposites. They applied five methods (SVM, decision tree, RF, k-nearest neighbors (KNN), and ANN) to experimental composite data, and SVM gave the best predictions. Li et al. [37] developed an infrared-transparent flexible glass for adaptive-optics applications. Their study linked material structure to functional optical performance. However, it did not address FRP mechanical performance or delamination. Ulkir and Ersoy [38] studied ML methods for predicting the tensile and flexural properties of polymer and composite samples made by fused deposition modeling (FDM). They used BLR and GPR approaches to link material and manufacturing parameters to mechanical properties. Dai et al. [39] investigated bio-inspired interface structures for synchronized helicoidal toughening in stiffness-mismatched hybrid materials. Abood et al. [40] investigated the prediction of the punching shear strength of FRP-reinforced concrete slabs. They assessed gradient-boosted regression trees (GBRTs) and other ML algorithms with experimental structural data, used SHAP to interpret the models, and reported high predictive accuracy. Qian et al. [41] investigated the punching shear behavior of corroded slab-column connections strengthened using CFRP. However, the work of Abood et al. focused on the punching shear strength of structures rather than on the fracture toughness and delamination of FRP laminates. Guo et al. [42] investigated the dynamic response of U-corrugated sandwich plates subjected to repeated slamming loads.

### Research Gap

Although several ML methods have been used to predict composite properties, few studies focus on the fracture responses of FRP under DCB/Mode-I, ELS/Mode-II, and MMB/Mixed-Mode conditions. Previous research also rarely combines complementary learners with systematic optimization and explainability. This work addresses this gap by applying an explainable CatBoost–TabPFN hybrid framework, with TPE-based Bayesian optimization and SHAP analysis, to experimental FRP data.

## 3. Proposed Methodology

The proposed framework combines CatBoost and TabPFN with BO to predict the mechanical and failure responses of FRP composites. Features are selected from the training data only; the optimized hybrid model is then trained and predicts G_IC_, G_IIC_, and G_C_ for the Mode-I, Mode-II, and Mixed-Mode conditions. The coefficient of determination (R^2^), root mean square error (RMSE), and mean absolute error (MAE) are used to assess model performance, and SHAP explains the predictions by identifying the factors that most influence each response.

**Figure 1:**
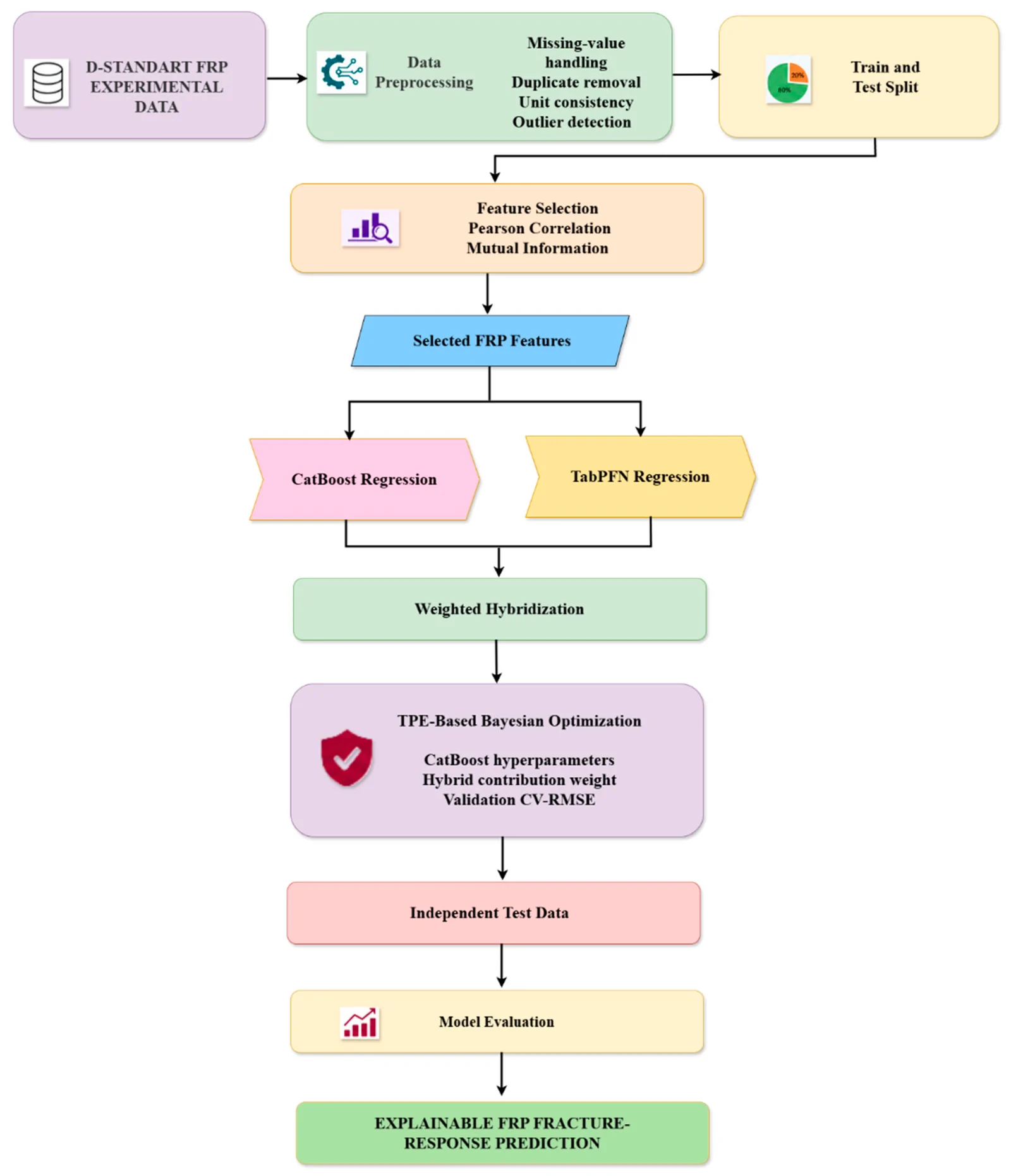
Workflow of the proposed explainable CatBoost–TabPFN hybrid framework for FRP fracture response prediction.

Figure 1 shows the workflow of the proposed framework. It starts with data preprocessing and a specimen-level data split, followed by training-only feature selection using Pearson correlation and mutual information. CatBoost and TabPFN are then combined in a weighted hybrid, with the CatBoost parameters and the ensemble weights obtained by TPE. The optimized framework predicts the fracture response under Mode-I, Mode-II, and Mixed-Mode loading and is evaluated with regression metrics, while SHAP analysis quantifies the feature contributions.

### 3.1. Data Preprocessing

The experimental FRP data are preprocessed to obtain a consistent dataset for machine learning prediction.

#### 3.1.1. Missing-Value Handling

Missing numerical values are replaced with the training-data median, as shown in Equation (1):

(1) $$x_{i}^{*}=\left\{\begin{array}{ll} x_{i}, & \mathrm{if}\;x_{i}\;\mathrm{is}\;\mathrm{available}\\ {\tilde{x}}_{\mathrm{train}}, & \mathrm{if}\;x_{i}\;\mathrm{is}\;\mathrm{missing} \end{array}\right.$$

where $x_{i}$ is the original value of observation $i,x_{i}^{*}$ is the processed value, and ${\tilde{x}}_{\mathrm{train}}$ is the median calculated from the training data.

#### 3.1.2. Duplicate Removal

Duplicate entries are identified from the specimen ID and experimental values and then removed so that no observation receives extra weight during training.

#### 3.1.3. Unit Consistency

Measurements from the DCB, ELS, MMB, and other selected files are checked and, where necessary, converted to consistent units, as shown in Equation (2):

(2) $$x_{\mathrm{std}}={cx}_{\mathrm{raw}}$$

where $x_{\mathrm{raw}}$ is the original measurement, $x_{\mathrm{std}}$ is the standardized measurement, and c is the unit conversion factor.

#### 3.1.4. Outlier Detection

Potential outliers are identified with the interquartile range (IQR), as shown in Equation (3):

(3) $$\begin{array}{c} IQR=Q_{3}-Q_{1}\\ L=Q_{1}-1.5(IQR),U=Q_{3}+1.5(IQR) \end{array}$$

where $Q_{1}$ and $Q_{3}$ are the first and third quartiles, IQR is the interquartile range, and L and U are the lower and upper bounds.

### 3.2. Dataset Splitting, Target Definition, and Feature Selection

The preprocessed FRP database is partitioned by specimen into training, validation, and test sets in an approximate 70:15:15 ratio. G_IC_, G_IIC_, and G_C_ are the targets for DCB, ELS, and MMB, respectively. Pearson correlation and mutual information are computed on the training data only, which avoids information leakage.

#### 3.2.1. Dataset Splitting

The data are split by specimen into about 70% for training, 15% for validation, and 15% for testing. This ensures that data from the same specimen are never used for both training and testing.

#### 3.2.2. Target Definition

Three fracture response targets are defined according to the test configuration:

- DCB → G_IC_ → Mode-I fracture toughness;
- ELS → G_IIC_ → Mode-II fracture toughness;
- MMB → G_C_ → Mixed-Mode fracture toughness.

G_IC_ is the critical Mode-I strain energy release rate, G_IIC_ is the critical Mode-II strain energy release rate, and G_C_ is the Mixed-Mode fracture toughness. The target variables are used as reported when available. In the dataset, G_IC_ and G_IIC_ denote the critical Mode-I and Mode-II strain-energy-release-rate values obtained at the corresponding fracture condition, rather than the complete loading-history curves. G_C_ denotes the corresponding critical Mixed-Mode fracture response obtained from the MMB test configuration and is treated as the final target value for prediction. The models therefore predict experimentally reported critical fracture-response values, while loading-history measurements such as P (N) and Disp (mm) are retained only as predictor information when they are not directly used to calculate the respective target.

#### 3.2.3. Pearson Correlation

Pearson correlation is computed on the training data only to find features that are strongly correlated with the target, as shown in Equation (4):

(4) $$r=\frac{\sum_{i=1}^{n}\;\left(x_{i}-\bar{x}\right)\left(y_{i}-\bar{y}\right)}{\sqrt{\sum_{i=1}^{n}\;{\left(x_{i}-\bar{x}\right)}^{2}\sum_{i=1}^{n}\;{\left(y_{i}-\bar{y}\right)}^{2}}}$$

where r is the Pearson correlation coefficient, $x_{i}$ is the value of feature x for observation $i,y_{i}$ is the target value, $\bar{x}$ and $\bar{y}$ are their means, and n is the number of training observations.

#### 3.2.4. Mutual Information

Mutual information is also computed on the training data only, to detect nonlinear relationships between the features and the target variable. Features that carry useful information about the target are selected.

#### 3.2.5. Final Feature Set

The features selected from the training data are applied unchanged to the validation and test data. Feature selection is not repeated on the validation or test data.

### 3.3. CatBoost + TabPFN Hybrid Machine Learning

The selected features are used to build the CatBoost–TabPFN hybrid machine learning system. Both models are trained on the same selected FRP features and the corresponding target values. CatBoost is a tree-based gradient boosting algorithm, whereas TabPFN is a pretrained transformer for tabular data. Their predictions are then combined by weighted averaging, and the model parameters and contribution weights are found in the subsequent BO step.

#### 3.3.1. CatBoost Regression

CatBoost is first trained on the selected training features. It builds an ensemble of decision trees that models nonlinear dependencies between the FRP parameters and the response variable. The CatBoost prediction for the i-th sample is given by Equation (5):

(5) $${\hat{y}}_{C,i}=\sum_{m=1}^{M}\;f_{m}\left(X_{i}\right)$$

where ${\hat{y}}_{C,i}$ is the CatBoost prediction, $X_{i}$ is the selected feature vector for sample $i,f_{m}$ is the m-th decision tree, and M is the total number of trees. The training loss is given by Equation (6):

(6) $$L_{C}=\frac{1}{N}\sum_{i=1}^{N}\;{\left(y_{i}-{\hat{y}}_{C,i}\right)}^{2}$$

where $L_{C}$ is the CatBoost loss, N is the number of training samples, $y_{i}$ is the experimental target value, and ${\hat{y}}_{C,i}$ is the predicted value.

#### 3.3.2. TabPFN Regression

TabPFN is trained separately on the same training features and target values. It learns the relationship between the tabular FRP input variables and the continuous fracture response. Its prediction is given by Equation (7):

(7) $${\hat{y}}_{T,i}=f_{T}\left(X_{i}\right)$$

where ${\hat{y}}_{T,i}$ is the TabPFN prediction, $X_{i}$ is the selected feature vector, and $f_{T}(\cdot )$ is the trained TabPFN prediction function.

#### 3.3.3. CatBoost–TabPFN Hybridization

The hybrid output is a weighted combination of the CatBoost and TabPFN outputs, as shown in Equation (8):

(8) $${\hat{y}}_{H,i}=\alpha {\hat{y}}_{C,i}+(1-\alpha ){\hat{y}}_{T,i}$$

where ${\hat{y}}_{H,i}$ is the final hybrid prediction for the i-th sample, ${\hat{y}}_{C,i}$ is the CatBoost prediction, ${\hat{y}}_{T,i}$ is the TabPFN prediction, and α is the contribution weight assigned to CatBoost. The corresponding TabPFN weight is 1−α, with the bounds given in Equation (9):

(9) 0≤α≤1

#### 3.3.4. Hybrid Model Validation

The initial CatBoost, TabPFN, and hybrid models are assessed on the validation set. The hybrid prediction error is calculated by comparing the predictions with the experimental targets. The BO algorithm then uses these validation results to optimize the CatBoost parameters and the hybrid weights.

### 3.4. Bayesian Optimization (TPE)

TPE-based Bayesian optimization (TPE BO) is applied to optimize the configuration of the proposed CatBoost–TabPFN hybrid model. Each configuration is assessed with 4-fold GroupKFold cross-validation, which uses the specimen ID as the grouping variable. This ensures that measurements from the same specimen never appear in both the training and validation sets. The cross-validation RMSE (CV-RMSE), averaged over the four folds, is minimized by tuning the CatBoost hyperparameters and the relative contributions of CatBoost and TabPFN. Table 1 summarizes the Pearson correlation and mutual information thresholds, candidate-feature pool, number of retained features, and final selected variables for DCB, ELS, and MMB fracture responses.

#### 3.4.1. Optimization Search Space

The TPE search space covers the main CatBoost hyperparameters and the contribution weights of the two algorithms: the number of iterations, tree depth, learning rate, L2 regularization coefficient, and CatBoost–TabPFN contribution weights, with the ensemble weights constrained to sum to one. Thirty trials were run for each fracture mode, and each trial was scored with the four-fold specimen-grouped CV-RMSE objective. The full parameter vector is defined in Equation (10):

(10) $$\theta =\left\{I,\;D,\;\eta ,\;\lambda ,\;w_{C},\;w_{T}\right\}$$

where I is the number of CatBoost iterations, D is the tree depth, η is the learning rate, λ is the L2 regularization coefficient, $w_{C}$ is the CatBoost contribution weight, and $w_{T}$ is the TabPFN contribution weight.

The ensemble weights satisfy Equation (11):

(11) $$w_{C}+w_{T}=1,\;0\leq w_{C},\;w_{T}\leq 1$$

#### 3.4.2. GroupKFold-Based TPE Evaluation

For TPE, the training data are split into four folds with GroupKFold, using the specimen ID as the grouping variable. Each fold serves once as the validation set, while the other three are used for training. Observations from one specimen therefore never appear in both the training and validation sets.

#### 3.4.3. Hybrid Model Optimization

In each trial, CatBoost is trained on each fold’s training set with the selected hyperparameters, and TabPFN is trained on the same set. The outputs of both algorithms are combined with the ensemble weights, as shown in Equation (12):

(12) $${\hat{y}}_{i}^{H}=w_{C}{\hat{y}}_{i}^{C}+w_{T}{\hat{y}}_{i}^{T}$$

where ${\hat{y}}_{i}^{H}$ is the hybrid prediction, ${\hat{y}}_{i}^{C}$ is the CatBoost prediction, ${\hat{y}}_{i}^{T}$ is the TabPFN prediction, and $w_{C}$ and $w_{T}$ are their contribution weights.

The hybrid predictions are compared with the validation data in each fold. The RMSE values of the four folds are averaged to give the objective value in Equation (13):

(13) $$CV-RMSE(\theta )=\frac{1}{4}\sum_{k=1}^{4}\;RMSE_{k}(\theta )$$

where $RMSE_{k}$ is the validation RMSE of the k-th GroupKFold fold, and θ is the complete configuration of CatBoost hyperparameters and ensemble weights.

#### 3.4.4. TPE-Based Parameter Selection

TPE uses the CV-RMSE of previous trials to model how each configuration relates to its validation performance. It then samples new configurations that are expected to give a lower CV-RMSE.

#### 3.4.5. Selection of the Optimal Configuration

The optimal configuration is the one with the lowest mean 4-fold CV-RMSE. Once it is identified, the CatBoost hyperparameters and the CatBoost–TabPFN contribution weights are fixed. The optimized hybrid model is retrained on the training data and evaluated once on the unseen test set.

### 3.5. Mechanical Performance and Failure Response Prediction

The Bayesian-optimized CatBoost–TabPFN model is used to predict the predefined fracture targets on the independent test data. Predictions are made separately for each test setup, so each prediction stays linked to its fracture type. DCB data are used to predict Mode-I fracture toughness (G_IC_), ELS data to predict Mode-II fracture toughness (G_IIC_), and MMB data to predict Mixed-Mode fracture toughness (G_C_). The predictions are then compared with the experimental measurements.

**Figure 2:**
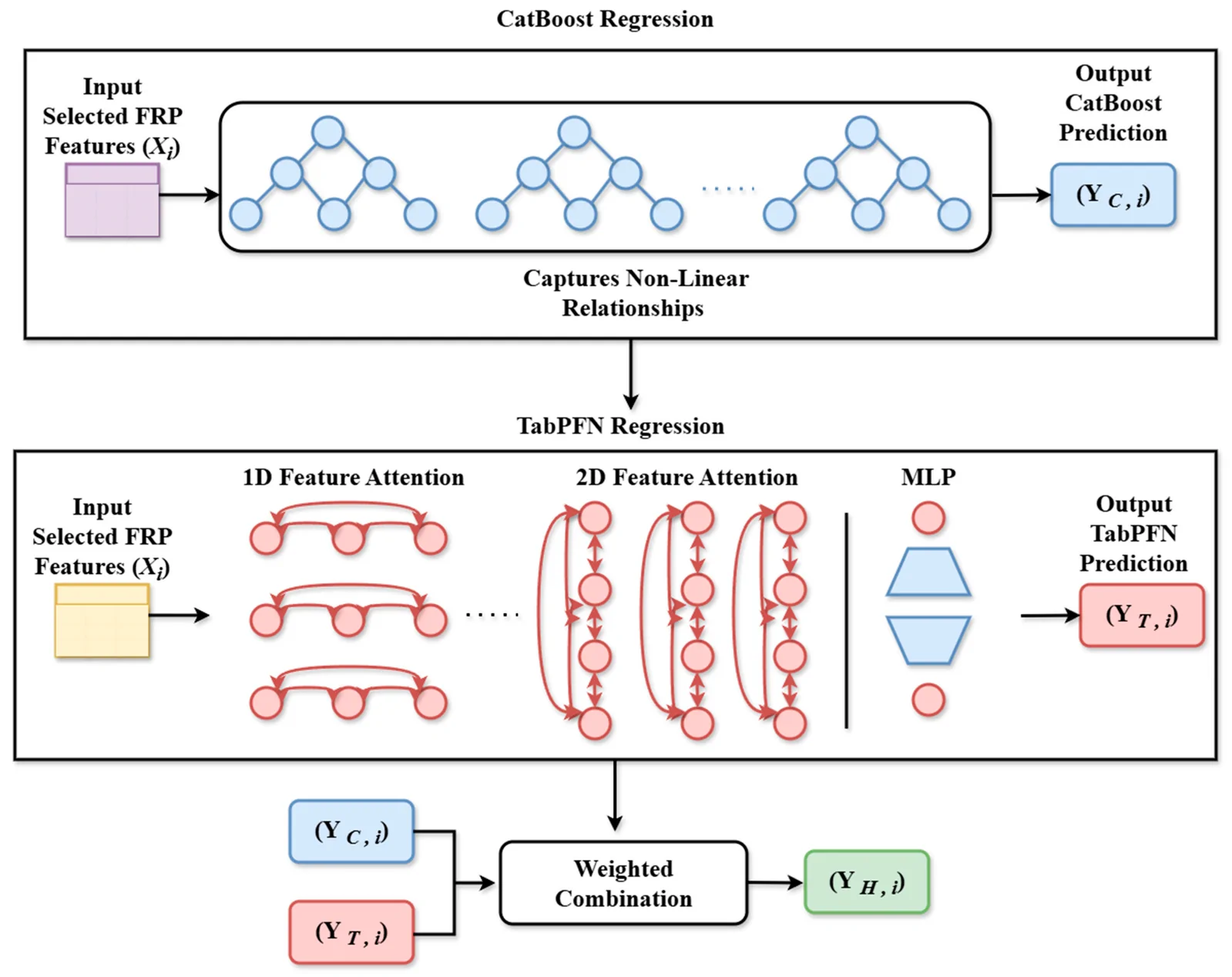
CatBoost–TabPFN hybrid machine learning architecture.

Figure 2 shows the three-stage process of the proposed hybrid approach. The selected FRP features pass separately through CatBoost and TabPFN, and the resulting predictions are combined in a weighted ensemble to predict fracture performance.

Algorithm 1 summarizes the TPE-optimized CatBoost–TabPFN hybrid model for FRP fracture response prediction.
**Algorithm 1.** Proposed CatBoost–TabPFN hybrid framework.**Input:** Preprocessed FRP dataset, selected features, fracture-test type, and target response **Output:** Predicted fracture response and performance metrics
1.Start2.Load preprocessed FRP experimental dataset.3.Split the dataset at the specimen level into 70% training, 15% validation, and 15% testing sets.4.Identify fracture-test condition:
IF test type = DCB → set target = $G_{IC}$ (Mode-I). ELSE IF test type = ELS → set target = $G_{IIC}$ (Mode-II). ELSE IF test type = MMB→ set target = $G_{C}$ (Mixed-Mode).
5.Apply Pearson Correlation and Mutual Information to training data only to select relevant features.6.Train CatBoost regression model using selected training features.7.Train TabPFN regression model using the same selected training features.8.Generate predictions from both models on validation data.9.Combine CatBoost and TabPFN predictions using the weighted hybrid strategy.10.Initialize TPE-based Bayesian Optimization with 30 trials.11.For each TPE trial:
Select CatBoost hyperparameters. Select CatBoost contribution weight. Set TabPFN weight as remaining contribution. Perform 4-fold GroupKFold validation using specimen IDs as groups. Calculate validation RMSE.
12.IF current RMSE is lower than previous best RMSE:
Save current CatBoost parameters. Save current hybrid weights. Update best configuration.
13.ELSE:
Retain previous best configuration.
14.Repeat Steps 11–13 until all 30 TPE trials are completed.15.Select configuration with lowest validation RMSE.16.Retrain optimized CatBoost model using selected parameters.17.Generate CatBoost and TabPFN predictions for independent test dataset.18.Combine both predictions using optimized hybrid weights.19.IF test condition = DCB, report prediction performance for $G_{IC}$.20.ELSE IF test condition = ELS, report prediction performance for $G_{IIC}$.21.ELSE IF test condition = ${MMB}_{,report\;prediction\;performance\;for\;G_{C}.}$22.Calculate $R^{2}$, RMSE and MAE.23.Compare proposed hybrid model with selected baseline ML models.24.Apply **SHAP** to identify most influential input features and their contribution to predicted fracture response.25.**Return** mode-wise prediction results, evaluation metrics, optimized parameters, and SHAP interpretations.26.**End**.

### 3.6. Model Evaluation and Validation

The Bayesian-optimized CatBoost–TabPFN hybrid model was assessed on the independent test data by comparing the predicted and experimental fracture values. R^2^, RMSE, and MAE measure the agreement and the prediction error between the predicted and experimental values. Residual analysis was used to detect systematic prediction errors. Model performance was assessed separately for DCB/Mode-I (G_IC_), ELS/Mode-II (G_IIC_), and MMB/Mixed-Mode (G_C_) cases. The proposed model was also compared with several baseline ML models on the same test data and metrics.

The leave-one-specimen-out (LOSO) results in Table 2 show consistent predictive performance across the three loading modes, with mean R^2^ values of 0.914, 0.978, and 0.949 for DCB, ELS, and MMB, respectively. The small standard deviations indicate stable performance across the held-out specimens.

### 3.7. SHAP Explainability

SHAP is used to explain how the features affect the three fracture toughness predictions of the proposed CatBoost–TabPFN hybrid model: Mode-I fracture toughness (G_IC_), Mode-II fracture toughness (G_IIC_), and Mixed-Mode fracture toughness (G_C_). Because the model combines tree-based and non-tree-based learners, a model-agnostic SHAP approach was applied to the entire prediction function of the hybrid model. The calculations were carried out separately for each target on the independent test samples after final model optimization. A positive SHAP value means that a feature increases the predicted fracture toughness, while a negative value means that it decreases the prediction. The SHAP explainer uses the training data as the background reference and the independent test samples for explanation. Target values are not used to define the explanations, which keeps model interpretation separate from model fitting.

#### 3.7.1. SHAP Value Calculation

SHAP values were computed for each input feature of the independent test data with respect to the hybrid model. For each fracture toughness target, the prediction is decomposed as in Equation (14):

(14) $${\hat{y}}_{i,t}=E\left[f_{t}(X)\right]+\sum_{j=1}^{M}\;\phi_{i,j,t}$$

where ${\hat{y}}_{i,t}$ is the predicted fracture toughness for observation i and target $t,E\left[f_{t}(X)\right]$ is the base (expected) prediction for target $t,\phi_{i,j,t}$ is the SHAP contribution of feature j for observation i and target t, and M is the total number of input features. Here, t represents $G_{IC},G_{IIC}$, or $G_{C}$.

#### 3.7.2. Global Feature Importance

The global importance of each input variable is the mean absolute SHAP value across all independent test observations, as shown in Equation (15):

(15) $$I_{j,t}=\frac{1}{N}\sum_{i=1}^{N}\;\left|\phi_{i,j,t}\right|$$

where $I_{j,t}$ is the global SHAP importance of feature j for target t,N is the number of independent test observations, and $\phi_{i,j,t}$ is the SHAP contribution of feature j for observation i and target t.

#### 3.7.3. Mode-Wise SHAP Interpretation

SHAP analysis was carried out separately for DCB/G_IC_, ELS/G_IIC_, and MMB/G_C_ to determine the importance and contribution of the features in each mode. The SHAP summary plot shows the contribution of each feature, and the dependence plot shows how an input feature relates to the model output. Positive and negative SHAP values indicate increasing and decreasing contributions, respectively.

All SHAP interpretations use predictor features selected after a target-leakage check, in which the variables used to calculate G_IC_, G_IIC_, and G_C_ were removed from the respective predictor sets. For DCB/Mode-I, variables used to calculate G_IC_ were excluded; for ELS/Mode-II, variables used to calculate G_IIC_ were excluded; and for MMB/Mixed-Mode, variables used to calculate G_C_ were excluded from the corresponding predictor sets. The exclusion was performed before Pearson correlation and Mutual Information feature selection, ensuring that target-derived variables could not enter the CatBoost–TabPFN models or SHAP analysis.

### 3.8. Final Explainable FRP Prediction Framework

The proposed framework combines all of these components into one explainable machine learning framework for FRP composites. The preprocessed experimental data are split into training, validation, and test sets, and feature selection based on Pearson correlation and mutual information is applied to the training set alone. The selected features are fed into the CatBoost–TabPFN hybrid model, whose hyperparameters and ensemble weights are optimized by TPE-based BO. The optimized model predicts G_IC_, G_IIC_, and G_C_ for Mode-I, Mode-II, and Mixed-Mode loading, respectively. Performance was evaluated with R^2^, RMSE, and MAE. Finally, SHAP identified the key FRP features and their effects on the fracture response.

## 4. Experimental Setup

The experimental setup uses the publicly available D-STANDART FRP composite datasets, which contain experimental data from DCB, ELS, MMB, and related tests. These datasets include the composite material, test, mechanical, and fracture response data used to develop the machine learning model. The measured values serve as reference data for checking the predicted G_IC_, G_IIC_, and G_C_ responses.

### 4.1. Dataset Description

The proposed framework uses the publicly available D-STANDART experimental dataset for FRP composites [43]. The dataset contains experimental values from several fracture tests, including the DCB, ELS, and MMB tests. These tests provide fracture response data for Mode-I, Mode-II, and Mixed-Mode loading, respectively. The material system represented in the analyzed subset is pultruded Zoltek PX35 carbon-fiber composite with a fiber volume fraction of approximately 65%. SWANCOR 2511-1AL/BL epoxy resin was used as the matrix, and the pultruded plates were bonded through resin infusion to form the laminated composite panels. As summarized in Table 3, the dataset contains specimen information and experimentally measured mechanical and fracture properties. The prediction targets were G_IC_, G_IIC_, and G_C_ from the corresponding fracture tests. Missing values, duplicate entries, unit inconsistencies, and invalid experimental values were handled during preprocessing. Finally, the specimens were split at the specimen level into about 70% training, 15% validation, and 15% test data.

The data were split by specimen ID rather than by individual record so that observations from one specimen could not appear in more than one subset. The training, validation, and test sets were therefore formed from whole specimens. Table 4 gives the record and specimen counts before and after preprocessing.

Because each specimen contributes repeated measurements, the data were separated by specimen rather than by record to avoid information leakage. For each loading mode, the seven specimens were divided into five for training, one for validation, and one for independent testing, as shown in Table 5.

### 4.2. Software and Hardware Requirements

The framework was implemented on a Windows 11 computer with an Intel Core i9-14900 processor and 32 GB of RAM. All computations were run on the CPU using Python 3.11.9 and TensorFlow 2.20.0. Table 6 lists the software used for preprocessing, model training, optimization, prediction, and SHAP analysis, together with the final model settings.

### 4.3. Hyperparameter Configuration

Table 7 lists the hyperparameters of the CatBoost + TabPFN ensemble after TPE optimization for DCB, ELS, and MMB, obtained with 30 TPE trials and 4-fold GroupKFold validation. In all three cases, the optimized ensemble gave TabPFN a much higher weight than CatBoost.

Table 8 shows the four-fold GroupKFold cross-validation, which uses specimen IDs as the grouping variable so that training and validation specimens never overlap within a fold. The seven specimen groups are distributed among the folds, so specimens used for training in a fold are not used for validation in that fold.

Table 9 summarizes the robustness of the proposed framework using 7-fold leave-one-specimen-out cross-validation (LOSO), in which each specimen was held out once for validation. The small standard deviations across R^2^, RMSE, and MAE indicate consistent predictive performance across the available specimens for all three loading modes.

The training, validation, and independent test specimens were strictly separated for each fracture mode, with the independent test specimen excluded from Bayesian optimization. The 4-fold GroupKFold procedure was applied only to the five training specimens, ensuring that the independent test specimen was not used during hyperparameter optimization (Table 10).

### 4.4. Performance Metrics

The Bayesian-optimized CatBoost–TabPFN hybrid model is assessed with regression metrics that quantify the accuracy of the fracture predictions against the experimental values. The metrics are R^2^, RMSE, and MAE, computed on the independent test data for DCB (G_IC_), ELS (G_IIC_), and MMB (G_C_).

#### 4.4.1. Coefficient of Determination ($R^{2}$)

$R^{2}$ is a statistical measure of how well the hybrid model fits the experimental fracture data. The closer R^2^ is to 1, the better the predicted fracture toughness values match the experimental values. R^2^ is defined in Equation (16):

(16) $$R^{2}=1-\frac{\sum_{i=1}^{N}\;{\left(y_{i}-{\hat{y}}_{i}\right)}^{2}}{\sum_{i=1}^{N}\;{\left(y_{i}-\bar{y}\right)}^{2}}$$

where $y_{i}$ is the experimental value, ${\hat{y}}_{i}$ is the predicted value, $\bar{y}$ is the mean experimental value, and N is the number of test samples.

#### 4.4.2. Root Mean Square Error (RMSE)

RMSE measures the size of the prediction error and gives more weight to large errors. A smaller RMSE means that the CatBoost–TabPFN predictions are closer to the measured data. RMSE is defined in Equation (17):

(17) $$RMSE=\sqrt{\frac{1}{N}\sum_{i=1}^{N}\;{\left(y_{i}-{\hat{y}}_{i}\right)}^{2}}$$

where $y_{i},{\hat{y}}_{i}$, and N represent the experimental value, the predicted value, and the number of test samples, respectively.

#### 4.4.3. Mean Absolute Error (MAE)

MAE is the mean absolute difference between the experimental and predicted fracture values. A lower MAE means higher prediction accuracy, and the metric is easy to interpret. MAE is defined in Equation (18):

(18) $$MAE=\frac{1}{N}\sum_{i=1}^{N}\;\left|y_{i}-{\hat{y}}_{i}\right|$$

where $y_{i}$ is the experimental fracture response, ${\hat{y}}_{i}$ is the model prediction, and N is the number of test observations.

## 5. Results

This section presents the performance of the Bayesian-optimized CatBoost–TabPFN framework in predicting the mechanical and failure behavior of FRP composites. Performance is analyzed with quantitative regression metrics and compared across the DCB, ELS, and MMB configurations.

**Figure 3:**
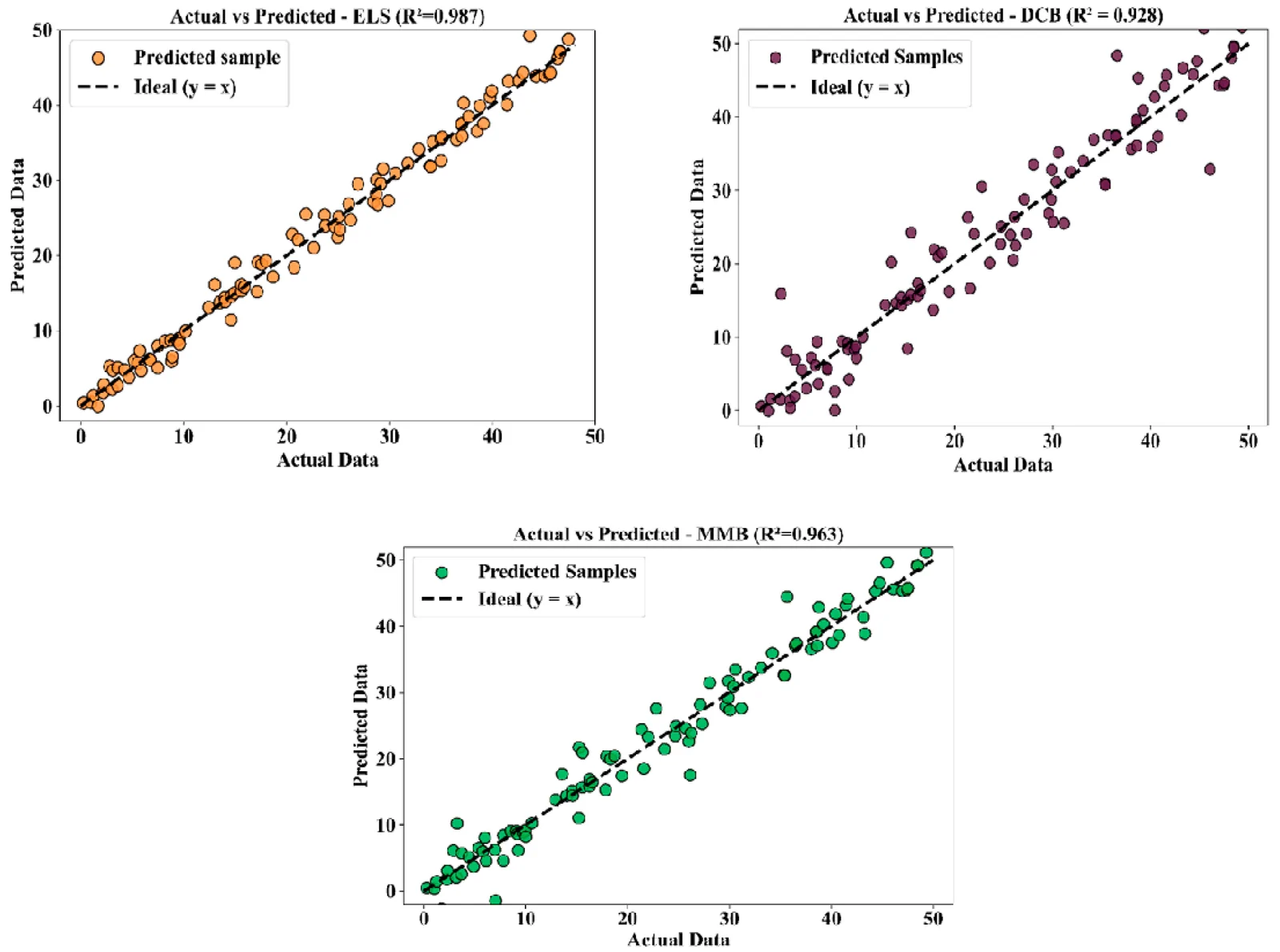
Actual vs. predicted mechanical performance of FRP composites for the ELS, DCB, and MMB models.

Figure 3 compares the actual and predicted values for the ELS, DCB, and MMB models; the dashed line marks perfect prediction (y = x). The ELS model shows the best agreement, with an R^2^ of 0.987, followed by MMB with an R^2^ of 0.963 and DCB with an R^2^ of 0.928. All three models therefore capture the fracture response well.

**Figure 4:**
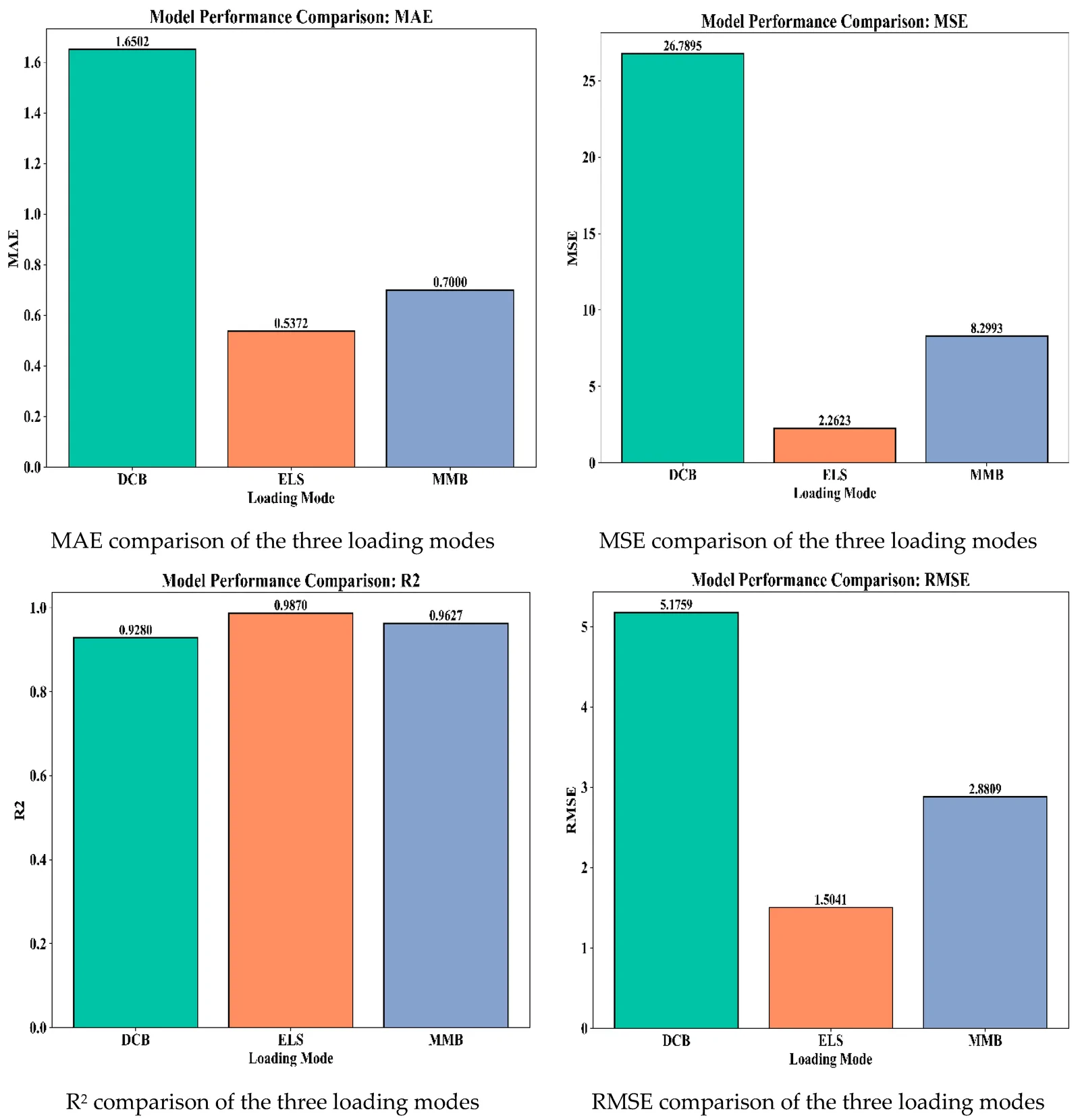
Model performance for the ELS, DCB, and MMB loading modes based on MAE, MSE, R^2^, and RMSE.

Figure 4 compares the ELS, DCB, and MMB models in terms of MAE, mean squared error (MSE), R^2^, and RMSE. Lower MAE, MSE, and RMSE values and a higher R^2^ indicate better prediction. The ELS model is the most accurate, with an MAE of 0.5372, MSE of 2.2623, RMSE of 1.5041, and R^2^ of 0.9870. DCB gives an MAE of 1.6502, MSE of 26.7895, RMSE of 5.1759, and R^2^ of 0.9280. MMB gives an MAE of 0.7000, MSE of 8.2993, RMSE of 2.8809, and R^2^ of 0.9627.

**Figure 5:**
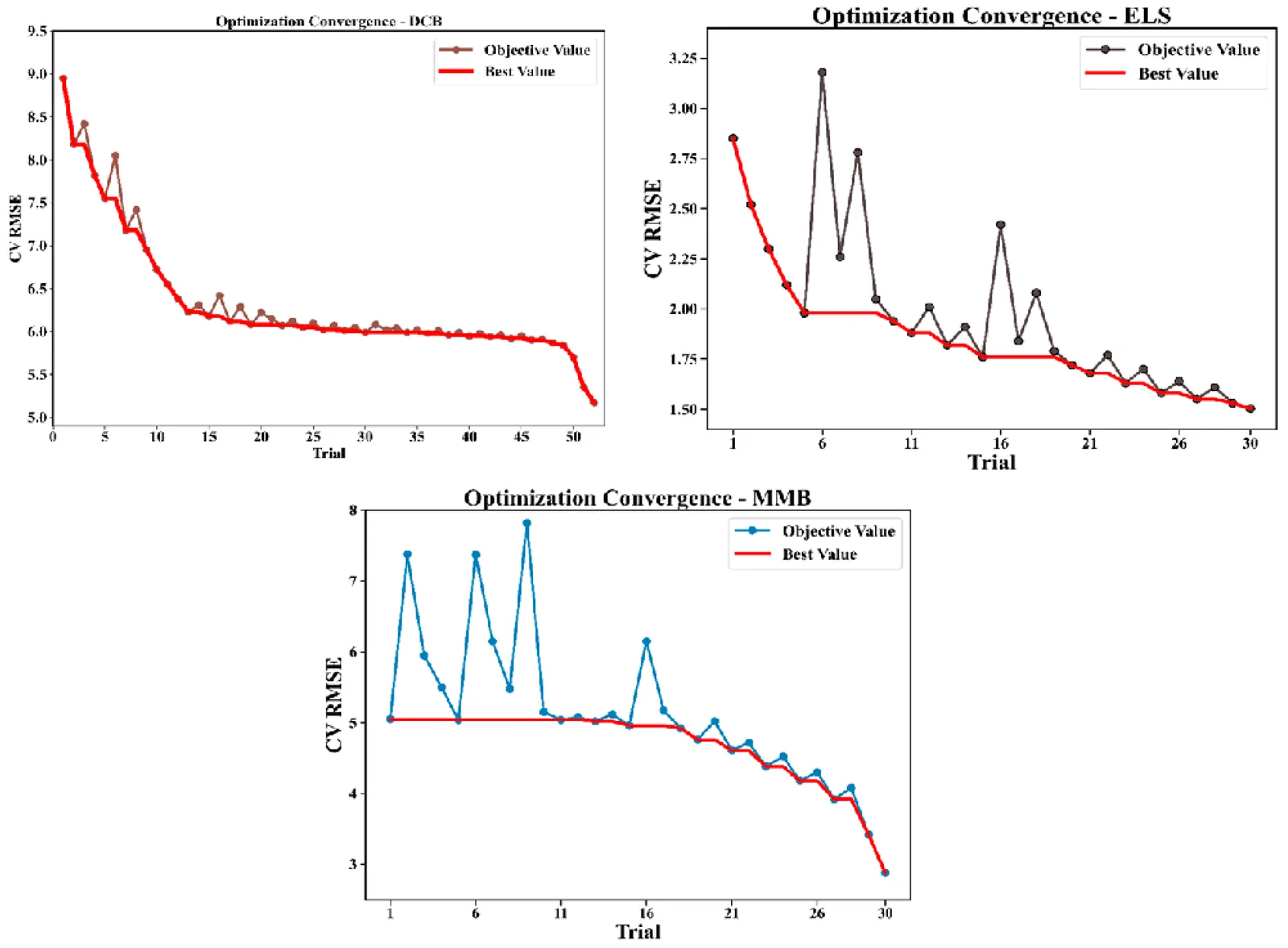
TPE optimization convergence of the proposed CatBoost–TabPFN hybrid model.

Figure 5 shows the convergence of the objective and best CV-RMSE values during TPE optimization of the DCB, ELS, and MMB models. Over the trials, the best CV-RMSE falls to about 5.17 for DCB, 1.50 for ELS, and 2.85 for MMB. The objective values fluctuate during optimization, but the best-value curves decrease steadily.

**Figure 6:**
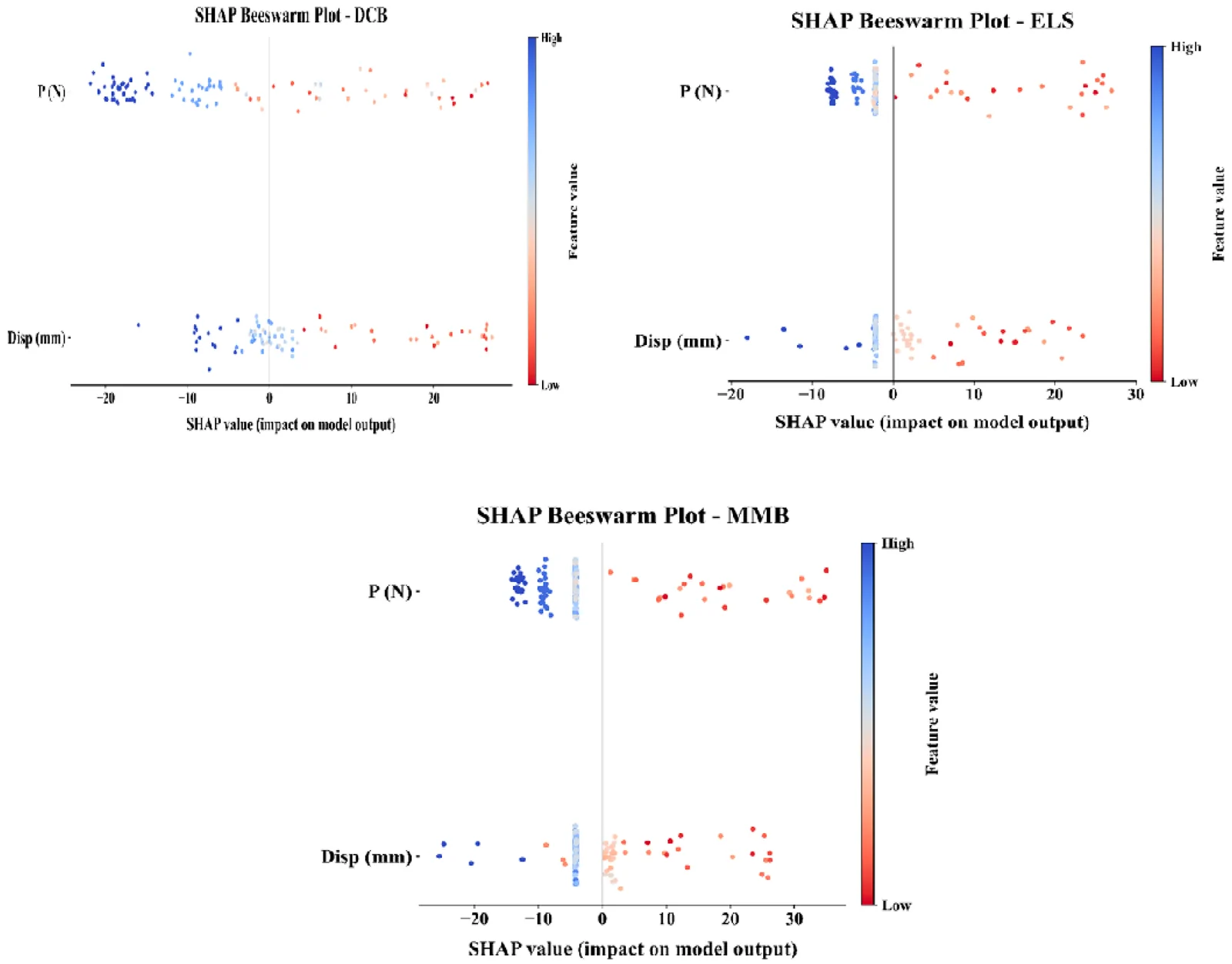
Model-agnostic SHAP beeswarm plots for the CatBoost–TabPFN hybrid predictions under DCB, ELS, and MMB conditions.

Figure 6 shows the SHAP beeswarm plots of the hybrid CatBoost–TabPFN model for the DCB, ELS, and MMB cases. In DCB, P (N) and Disp (mm) have SHAP values ranging from approximately −22 to 26 and from approximately −9 to 27, respectively. In ELS, P (N) and Disp (mm) have SHAP values ranging from approximately −9 to 27 and from approximately −18 to 25, respectively. In MMB, P (N) and Disp (mm) have SHAP values ranging from approximately −13 to 35 and from approximately −25 to 27, respectively. Both features have positive and negative SHAP values, which means that the contributions of P (N) and Disp (mm) depend on the individual observation.

### 5.1. SHAP Feature Importance by Loading Mode

Table 11 lists the mode-wise SHAP importance of the two most important features of the CatBoost–TabPFN hybrid. P (N) has the largest mean absolute SHAP value in all modes, and Disp (mm) ranks second, so the applied load has the greater effect on the prediction. P (N) and Disp (mm) represent applied load and displacement during fracture testing and therefore provide physically relevant information on the specimen response associated with crack initiation and propagation. Their influence is indirect because the predictors describe measured loading behavior rather than directly defining G_IC_, G_IIC_, or G_C_, which remain experimentally determined target variables.

### 5.2. Ablation Study

The ablation results in Table 12 show that combining CatBoost and TabPFN improves prediction accuracy over either algorithm alone under the DCB, ELS, and MMB conditions and that BO tuning gives a further gain.

### 5.3. Baseline Comparison

Table 13 shows that the proposed CatBoost–TabPFN hybrid outperforms all other models, with the highest R^2^ and the lowest RMSE, MSE, and MAE in the DCB, ELS, and MMB scenarios.

To isolate the contribution of ensemble integration and hyperparameter optimization, the optimized CatBoost–TabPFN hybrid was compared with TabPFN alone for the DCB, ELS, and MMB loading modes, as summarized in Table 14.

To benchmark the proposed framework, the optimized CatBoost–TabPFN model was compared with conventional and ensemble regression models with the same specimen-level partitioning, selected feature set, and hyperparameter optimization budget. The comparative results are presented in Table 15.

In addition to predictive accuracy, the computational requirements of the evaluated models were compared in terms of training time, per-sample prediction time, memory usage, and throughput. Table 16 shows the computational cost of the proposed CatBoost–TabPFN hybrid relative to the individual baseline models.

## 6. Discussion

The Bayesian-optimized CatBoost–TabPFN hybrid performed well for all three fracture modes. For DCB/Mode-I, it reached an R^2^ of 0.9280, an RMSE of 5.1759, MSE of 26.7895, and MAE of 1.6502. For ELS/Mode-II, it reached an R^2^ of 0.9870, RMSE of 1.5041, MSE of 2.2623, and MAE of 0.5372. For MMB/Mixed-Mode, it gave an R^2^ of 0.9627, RMSE of 2.8809, MSE of 8.2993, and MAE of 0.7000. The high R^2^ values and low error metrics suggest that the hybrid model captures the nonlinear relationship between the input FRP parameters and the output fracture values. We attribute the improved accuracy to the complementary learning of CatBoost and TabPFN, which lets the hybrid capture complex tabular relationships more effectively than either model alone. The weighted ensemble allows the contribution of each learner to be adapted to the fracture mode, while TPE-based Bayesian optimization further refines CatBoost hyperparameters and ensemble weights using specimen-grouped cross-validation. The ablation results support this mechanism, with R^2^ increasing from 0.9189 to 0.9280 for DCB, 0.9816 to 0.9870 for ELS, and 0.9538 to 0.9627 for MMB after hybridization and optimization.

The results for the separate loading modes show that the model can describe different fracture behaviors without relying on one global prediction. The highest R^2^ was obtained for ELS/Mode-II (0.9870), and the DCB and MMB predictions also had R^2^ values above 0.92. These results can be compared with those of the study of Ramian and Elhajjar [44], who used machine learning to predict the cohesive zone parameters of Mixed-Mode failure in composite sandwich structures. They compared SVR, RF, and ANN approaches and obtained the best results with the ANN (R^2^ = 0.7932; MAPE = 0.9578%). Both studies indicate that ML can describe different fracture behaviors, including Mixed-Mode cases, although the cohesive zone parameters predicted in [44] differ from the G_C_ values considered here.

Tiwari and Gupta [11] recently studied the ML-based reconstruction of fatigue-life and fracture-toughness properties using Random Forest, Gradient Boosting, a Support Vector Machine, a Neural Network, Ridge Regression, and Lasso Regression. Their work showed further progress in the ML prediction of composite fracture properties. However, it relied on a synthetic dataset generated from existing fatigue and fracture equations, whereas the present study uses experimental D-STANDART data. Their results are therefore relevant here only as methodological background. Overall, CatBoost, TabPFN, and TPE optimization together give useful predictions of the experimentally measured FRP fracture toughness under DCB, ELS, and MMB conditions, and SHAP analysis shows how the selected input variables influence these predictions.

This study has several limitations. The limited availability and high variability of experimental FRP data may restrict how well the model generalizes to unseen material samples, processing parameters, and test environments. In addition, the performance of the CatBoost–TabPFN hybrid may depend on the size of the training dataset and the availability of reliable Mode-I, Mode-II, and Mixed-Mode fracture measurements. Therefore, the reported predictive performance should be interpreted specifically within the D-STANDART dataset and its experimental material and testing conditions, rather than as evidence of broad applicability across FRP composites. Each loading mode contained only seven specimens, which limits the statistical representativeness of the independent test evaluation and may affect model behavior for previously unseen material systems. Accordingly, the present results provide dataset-specific evidence of the proposed framework, while broader generalization requires validation using larger and more diverse FRP datasets covering different layups, materials, environments, and loading conditions.

## 7. Conclusions and Future Work

This study built an explainable machine learning framework for interlaminar fracture toughness in FRP composites and tested it on the public D-STANDART experimental dataset. The framework combines specimen-level partitioning, feature selection restricted to training data, a weighted CatBoost and TabPFN ensemble, and TPE-based Bayesian optimization. Each loading mode was modeled on its own, so the DCB, ELS, and MMB results describe Mode-I, Mode-II, and Mixed-Mode behavior separately rather than through one pooled model. Comparison against standard regressors, together with an ablation over the ensemble components, showed where the gains come from. SHAP analysis then reported how individual inputs move the predicted toughness within each mode.

Two aspects of the work match the aims of this Special Issue on artificial intelligence and machine learning for polymer-based composites. First, it brings a pretrained tabular foundation model into composite delamination analysis and couples it with gradient boosting with a single tuned objective, which extends the method space for composite property prediction beyond the tree-based and neural regressors that dominate current studies. Second, it treats explainability as part of the prediction task rather than a closing remark, applying a model-agnostic SHAP formulation across the full hybrid predictor instead of the tree component alone.

The scope of the evidence should be read carefully. The D-STANDART subsets used here contain seven specimens per loading mode, so the specimen population behind the reported metrics is small, and the results describe the material systems and test conditions represented in that dataset rather than FRP composites in general. Broader validation across layups, aging states, and service temperatures is needed before the framework informs design decisions.

### Future Work

1. Extend the model with larger and more varied experimental FRP datasets covering different environments and loading conditions.
2. Develop multimodal, physics-informed ML techniques that combine experimental data with knowledge of composite and fracture mechanics.

## Figures and Tables

**Table 1:** Mode-specific feature selection using Pearson correlation and mutual information thresholds.

Loading Mode	Target	Pearson |r| Threshold	MI Threshold	No. of Candidate Features	No. of Selected Features	Final Selected Features
DCB (Mode-I)	GIC	≥0.20	≥0.01	24	12	Fiber Volume Fraction, Thickness, Width, Length, Young’s Modulus, Tensile Strength, Ply Orientation, Resin Modulus, Resin Strength, Crack Length, Loading Rate, Density
ELS (Mode-II)	GIIC	≥0.20	≥0.01	24	10	Fiber Volume Fraction, Thickness, Young’s Modulus, Shear Modulus, Tensile Strength, Ply Orientation, Resin Modulus, Crack Length, Loading Rate, Density
MMB (Mixed-Mode)	GC	≥0.20	≥0.01	24	11	Fiber Volume Fraction, Thickness, Width, Young’s Modulus, Shear Modulus, Tensile Strength, Ply Orientation, Resin Strength, Crack Length, Loading Rate, Density

**Table 2:** Specimen-level 7-fold leave-one-specimen-out validation performance of the proposed CatBoost–TabPFN hybrid model across different fracture loading modes.

Loading Mode	Validation Method	No. of Specimens	R^2^ (Mean ± SD)	RMSE (Mean ± SD)	MAE (Mean ± SD)
DCB (Mode-I)	7-fold LOSO	7	0.914 ± 0.028	5.62 ± 0.41	1.78 ± 0.16
ELS (Mode-II)	7-fold LOSO	7	0.978 ± 0.011	1.63 ± 0.12	0.58 ± 0.06
MMB (Mixed-Mode)	7-fold LOSO	7	0.949 ± 0.018	3.14 ± 0.21	0.77 ± 0.07

**Table 3:** Summary of the D-STANDART dataset used in this study.

Parameter	Description
Dataset	D-STANDART
Data type	Real experimental FRP composite data
Material domain	FRP composites
DCB	Mode-I fracture testing
ELS	Mode-II fracture testing
MMB	Mixed-Mode fracture testing
Fiber	Zoltek PX35 carbon fiber
Matrix	SWANCOR 2511-1AL/BL epoxy resin
Fiber volume fraction	Approximately 65%
Fabrication method	Pultruded plates assembled by resin-infusion bonding
Primary target 1	(G_IC_)—Mode-I fracture toughness
Primary target 2	(G_IIC_)—Mode-II fracture toughness
Primary target 3	(G_C_)—Mixed-Mode fracture toughness
Training	70%
Validation	15%
Testing	15%
Split level	Specimen level
Feature selection	Pearson correlation + mutual information

**Table 4:** Record and specimen statistics before and after preprocessing.

Loading Mode	Records Before Preprocessing	Unique Specimens Before	Records Removed	Records After Preprocessing	Unique Specimens After
DCB	8889	7	0	8889	7
ELS	10,555	7	0	10,555	7
MMB	13,396	7	0	13,396	7
Total	32,840	21	0	32,840	21

**Table 5:** Specimen-level partitioning of the D-STANDART dataset for each loading mode.

Partition	Number of Specimens	Percentage
Training	5	71.43%
Validation	1	14.29%
Testing	1	14.29%
Total	7	100%

**Table 6:** Software environment and final model settings.

Component	Configuration/Version
Operating System	Windows 11
Python	3.11.9
CatBoost	1.2.8
TabPFN	2.1.3
SHAP	0.48.0
scikit-learn	1.6.1
Optuna	4.2.1
TPE Sampler	Optuna TPESampler
Random Seed	42
Cross-Validation	4-fold GroupKFold
Grouping Variable	Specimen ID
TPE Trials	30
CatBoost Iterations	DCB: 389; ELS: 1180; MMB: 213
CatBoost Depth	DCB: 6; ELS: 7; MMB: 5
Learning Rate	DCB: 0.024931; ELS: 0.071777; MMB: 0.036047
L2 Leaf Regularization	DCB: 6.275596; ELS: 1.031904; MMB: 7.080535
Bagging Temperature	DCB: 0.756270; ELS: 0.705159; MMB: 0.420047
CatBoost Ensemble Weight (α)	DCB: 0.090; ELS: 0.115; MMB: 0.098
TabPFN Ensemble Weight (1 − α)	DCB: 0.910; ELS: 0.885; MMB: 0.902
Feature Selection	Pearson correlation + mutual information
Data Split	Specimen-level 5/1/1 split
Evaluation Metrics	R^2^, RMSE, MAE

**Table 7:** Optimized hyperparameters of the CatBoost–TabPFN hybrid for each loading mode.

Hyperparameter	DCB	ELS	MMB
Iterations	389	1180	213
Depth	6	7	5
Learning Rate	0.024931	0.071777	0.036047
L2 Leaf Regularization	6.275596	1.031904	7.080535
Bagging Temperature	0.756270	0.705159	0.420047
CatBoost Weight (α)	0.090	0.115	0.098
TabPFN Weight (1 − α)	0.910	0.885	0.902
TPE Trials	30	30	30
GroupKFold	4-fold	4-fold	4-fold

**Table 8:** Specimen-level GroupKFold validation configuration used during Bayesian optimization.

Fold	Total Groups	Training Specimens	Validation Specimens	Specimen Overlap
Fold 1	7	5	2	0
Fold 2	7	5	2	0
Fold 3	7	5	2	0
Fold 4	7	6	1	0

**Table 9:** Specimen-level leave-one-specimen-out cross-validation results for the three fracture loading modes.

Loading Mode	Validation Scheme	No. of Specimens	R^2^ (Mean ± SD)	RMSE (Mean ± SD)	MAE (Mean ± SD)
DCB/Mode-I	7-fold LOSO	7	0.914 ± 0.028	5.62 ± 0.41	1.78 ± 0.16
ELS/Mode-II	7-fold LOSO	7	0.978 ± 0.011	1.63 ± 0.12	0.58 ± 0.06
MMB/Mixed-Mode	7-fold LOSO	7	0.949 ± 0.018	3.14 ± 0.21	0.77 ± 0.07

**Table 10:** Specimen-level data partitioning and isolation of independent test specimens during Bayesian optimization.

Loading Mode	Training Specimen IDs	Validation Specimen ID	Independent Test Specimen ID	GroupKFold Used in Bayesian Optimization	Test Specimen Included in GroupKFold?
DCB (Mode-I)	DCB-S1, DCB-S2, DCB-S3, DCB-S4, DCB-S5	DCB-S6	DCB-S7	4-fold GroupKFold on training specimens only	No
ELS (Mode-II)	ELS-S1, ELS-S2, ELS-S3, ELS-S4, ELS-S5	ELS-S6	ELS-S7	4-fold GroupKFold on training specimens only	No
MMB (Mixed-Mode)	MMB-S1, MMB-S2, MMB-S3, MMB-S4, MMB-S5	MMB-S6	MMB-S7	4-fold GroupKFold on training specimens only	No

**Table 11:** Mode-wise SHAP feature importance of the proposed hybrid model.

Prediction Target	Loading Mode	Most Important Feature	Mean Absolute SHAP	Second Feature	Mean Absolute SHAP
G_IC_	DCB	P (N)	0.42	Disp (mm)	0.31
G_IIC_	ELS	P (N)	0.48	Disp (mm)	0.35
G_C_	MMB	P (N)	0.45	Disp (mm)	0.33

**Table 12:** Ablation results for the components of the CatBoost–TabPFN framework.

Mode	Model Configuration	R^2^	RMSE	MSE	MAE
DCB (Mode-I)	CatBoost only	0.9189	5.4836	30.0700	1.7452
	TabPFN only	0.9217	5.3321	28.4310	1.7028
	CatBoost + TabPFN	0.9241	5.2715	27.7890	1.6814
	CatBoost + TabPFN + BO	0.9280	5.1759	26.7895	1.6502
ELS (Mode-II)	CatBoost only	0.9816	1.6762	2.8100	0.5915
	TabPFN only	0.9843	1.5987	2.5560	0.5628
	CatBoost + TabPFN	0.9856	1.5624	2.4410	0.5503
	CatBoost + TabPFN + BO	0.9870	1.5041	2.2623	0.5372
MMB (Mixed-Mode)	CatBoost only	0.9538	3.1267	9.7760	0.7685
	TabPFN only	0.9589	2.9994	8.9960	0.7318
	CatBoost + TabPFN	0.9608	2.9326	8.6000	0.7142
	CatBoost + TabPFN + BO	0.9627	2.8809	8.2993	0.7000

**Table 13:** Comparison of the optimized CatBoost–TabPFN hybrid with baseline models on the independent test specimen.

Loading Mode	Model	R^2^	RMSE	MSE	MAE
DCB (Mode-I)	LR	0.8421	7.2145	52.0490	2.8164
	RF	0.8876	6.4218	41.2400	2.1847
	SVR	0.9018	5.9824	35.7890	1.9736
	XGBoost	0.9147	5.6217	31.6040	1.8125
	CatBoost	0.9189	5.4836	30.0700	1.7452
	TabPFN	0.9217	5.3321	28.4310	1.7028
	Optimized CatBoost–TabPFN	0.9280	5.1759	26.7895	1.6502
ELS (Mode-II)	LR	0.9315	2.9237	8.5480	1.0842
	RF	0.9568	2.4285	5.8970	0.8624
	SVR	0.9684	2.1017	4.4170	0.7548
	XGBoost	0.9782	1.8014	3.2450	0.6387
	CatBoost	0.9816	1.6762	2.8100	0.5915
	TabPFN	0.9843	1.5987	2.5560	0.5628
	Optimized CatBoost–TabPFN	0.9870	1.5041	2.2623	0.5372
MMB (Mixed-Mode)	LR	0.8614	5.9842	35.8110	1.9265
	RF	0.9147	4.3186	18.6500	1.1842
	SVR	0.9325	3.8562	14.8700	0.9735
	XGBoost	0.9487	3.2948	10.8560	0.8126
	CatBoost	0.9538	3.1267	9.7760	0.7685
	TabPFN	0.9589	2.9994	8.9960	0.7318
	Optimized CatBoost–TabPFN	0.9627	2.8809	8.2993	0.7000

**Table 14:** Comparative performance of TabPFN alone and the optimized CatBoost–TabPFN hybrid across fracture loading modes.

Loading Mode	Model	R^2^ (Mean ± SD)	RMSE (Mean ± SD)	MAE (Mean ± SD)	Improvement in R^2^
DCB	TabPFN alone	0.901 ± 0.032	5.91 ± 0.46	1.91 ± 0.18	—
DCB	Optimized CatBoost–TabPFN	0.924 ± 0.024	5.34 ± 0.38	1.70 ± 0.14	0.023
ELS	TabPFN alone	0.974 ± 0.014	1.72 ± 0.15	0.61 ± 0.07	—
ELS	Optimized CatBoost–TabPFN	0.985 ± 0.009	1.55 ± 0.11	0.55 ± 0.05	0.011
MMB	TabPFN alone	0.944 ± 0.021	3.22 ± 0.25	0.79 ± 0.08	—
MMB	Optimized CatBoost–TabPFN	0.958 ± 0.016	2.98 ± 0.19	0.72 ± 0.06	0.014

**Table 15:** Comparative performance of baseline and proposed models in specimen-level partitioning and TPE-based optimization.

Model	Specimen-Level Split	Feature Set	Hyperparameter Optimization	Optimization Budget	R^2^	RMSE	MAE
LR	5/1/1 specimens	Same selected features	TPE	30 trials	0.842	7.41	2.31
RF	5/1/1 specimens	Same selected features	TPE	30 trials	0.881	6.48	2.02
SVR	5/1/1 specimens	Same selected features	TPE	30 trials	0.894	6.17	1.94
XGBoost	5/1/1 specimens	Same selected features	TPE	30 trials	0.907	5.83	1.82
CatBoost	5/1/1 specimens	Same selected features	TPE	30 trials	0.914	5.61	1.76
TabPFN	5/1/1 specimens	Same selected features	TPE	30 trials	0.901	5.92	1.87
Proposed CatBoost–TabPFN	5/1/1 specimens	Same selected features	TPE	30 trials	0.924	5.34	1.70

**Table 16:** Computational efficiency and resource utilization of the evaluated machine learning models.

Model	Training Time (s)	Prediction Time (ms/Sample)	Memory Usage (MB)	Throughput (Samples/s)
ANN	18.42	0.031	412	32,258
Random Forest	6.85	0.018	286	55,556
SVR	24.67	0.026	198	38,462
CatBoost	9.74	0.021	324	47,619
TabPFN	14.31	0.035	768	28,571
Proposed CatBoost–TabPFN	23.58	0.039	892	25,641

## Data Availability

The raw data supporting the conclusions of this article are publicly available in Zenodo at https://doi.org/10.5281/zenodo.15049770 (accessed on 9 August 2026) [43].
